# Safety and efficacy of stereotactic ablative brachytherapy as a salvage therapy for recurrent chest wall cancer: A retrospective, multicenter study

**DOI:** 10.3389/fonc.2022.957497

**Published:** 2023-02-07

**Authors:** Bin Huo, Zhe Ji, Chuang He, Wanying Yang, Yanli Ma, Xiaodong Huo, Zhe Wang, Xinxin Zhao, Jinchao Dai, Haitao Wang, Guanglie Chen, Ruoyu Wang, Yuqing Song, Kaixian Zhang, Xuequan Huang, Shude Chai, Junjie Wang

**Affiliations:** ^1^ Department of Oncology, The Second Hospital of Tianjin Medical University, Tianjin, China; ^2^ Department of Radiation Oncology, Peking University Third Hospital, Beijing, China; ^3^ Center of Minimally Invasive Intervention, Southwest Hospital of Army Medical University, Chongqing, China; ^4^ Department of Oncology, Tengzhou Central People’s Hospital, Tengzhou, China; ^5^ Department of Oncology, Staff Hospital of Chengde Iron and Steel Group Co. Ltd., Chengde, China; ^6^ Department of Radiation Oncology, Affiliated Zhongshan Hospital of Dalian University, Dalian, China; ^7^ Department of Oncology Radiotherapy, The First People's Hospital of Kerqin District, Tongliao, China; ^8^ Department of Nuclear Medicine, Qingdao Central Hospital, Qingdao, China

**Keywords:** recurrent chest wall cancer, stereotactic ablative brachytherapy, reirradiation, external beam radiotherapy (EBRT), salvage therapy

## Abstract

**Purpose:**

To evaluate the safety and efficacy of stereotactic ablative brachytherapy (SABT) as a salvage therapy for patients with recurrent chest wall cancer (rCWC) who have previously received external beam radiotherapy (EBRT) or surgery.

**Materials and methods:**

Between November 2013 and October 2020, a total of 130 patients (including 75 men with a median age of 63 years) with rCWC treated with SABT were enrolled in this multicenter retrospective study. There were 97 cases of non-small-cell lung carcinoma, 24 cases of breast cancer, and 9 cases of thymic cancer. Of the patients included, 102 patients previously received surgery and 58 patients received EBRT, with systemic treatment progressing after recurrence. None of them were suitable or refused to undergo salvage EBRT or surgery again.

**Results:**

During the 22 (4–70)-month median patient follow-up, 59 patients died. The local control (LC) rates at 6, 12, 24, and 36 months were 88.3%, 74.3%, 50.4%, and 36.7%, respectively. The 1-, 2- and 3-year survival rates were 85%, 56%, and 42%, respectively. The median overall survival was 26 months (95% CI, 18.9–33.1 months). The pain relief rate was 81%, and the median to remission time was 10 days. Univariate and multivariate analyses showed that independent prognostic factors for LC included tumor size and postoperative D90. On the other hand, independent prognostic factors for survival include the Karnofsky performance status (KPS) score, tumor size, and D90 19 patients (14.6%) developed grade I/II skin reaction complications. No grade III or severer complications occurred.

**Conclusion:**

SABT is safe and effective as a salvage therapy for rCWC following EBRT/surgery. For patients with a KPS score greater than 80, prescribed dose greater than 130 Gy, and tumor size less than 4 cm may bring better results.

## Introduction

Chest wall cancer (CWC) can arise from a variety of tumors, including breast cancer, lung cancer, mesothelioma, sarcoma, and thymic cancer. The primary treatment was surgical resection, but 50% of patients relapsed after surgery or radiotherapy (1–3). Once systemic therapy (chemotherapy or targeted therapy) progressed, the treatment options were limited; 5-year survival was only 17% (4–6). Retreatment with EBRT is promising, although the recurrence of the lesion within the previously irradiated field remains to be resolved. Hence, only well-selected patients can be considered for EBRT with higher toxicity (7, 8). Brachytherapy (BT) has the exceptional ability to deliver extremely high doses that external beam radiotherapy (EBRT) could never achieve within treated lesions, with the added benefit that doses drop off rapidly outside the target lesion by minimizing the exposure of uninvolved surrounding normal tissue (9).Stereotactic ablative BT (SABT) was designed to improve the ablative effect of radiation, which was achieved *via* improved image guidance and the calculation of ablative dose, shorter treatment duration, and better organ preservation (10). Recently collected data suggest that SABT has been shown to be safe and effective in the treatment of head and neck, thoracic, abdominal, retroperitoneal, and vertebral cancers, particularly for locally advanced or recurrent solid cancers following EBRT (11–15). Several single-institution retrospective studies of recurrent CWC (rCWC) patients have been published previously (16–18). Here, our multicenter retrospective study provided the long-term survival outcome data in rCWC patients treated with SABT and the data for real-world clinical practice.

## Materials and methods

### Patient selection criteria

This was a retrospective study, and 130 patients were enrolled [75 men and 55 women; the median age of 63 (32–85) years was related to rCWC receiving SABT treatment at eight medical centers in China between November 2013 and October 2020. There were 97 cases of non-small-cell lung carcinoma (40 cases of squamous cell carcinoma and 57 cases of adenocarcinoma), 24 cases of breast cancer, and 9 cases of thymic cancer. Of the patients included, 102 patients previously received surgery and 58 patients received EBRT, with systemic treatment progressing after recurrence and none of whom were candidates for or refused salvage surgery and/or repeat EBRT. The study enrolled patients with squamous cell lung cancer or driver-negative adenocarcinoma of the lung who had failed previous multiline chemotherapy. The driver-positive adenocarcinoma of the lung, who had failed previous targeted therapies. The characteristics of the patients are listed in **Table 1**. We analyzed several indicators such as the local control (LC) rate, progression-free survival (PFS), overall survival (OS), and complications. We evaluated tumor response according to Response Evaluation Criteria in Solid Tumors (RECIST) v1.1 (19).. Local disease control included complete response, partial response, and stable disease. Time to tumor progression from the SABT procedure was defined as time to progression. The time from SABT surgery to death from any cause or last follow-up was defined as OS. Complications were identified by Common Terminology Criteria for Adverse Events (CTCAE) v4.0 (CTCA) (20).

**Table 1:** Clinical characteristics of the 130 patients.

	n	%
Gender		
Male	75	57.7
Female	55	42.3
Median age (years)	63	
<70	92	70.8
≥70	38	29.2
KPS		
<80	59	45.4
≥80	71	57.7
Primary tumor		
Lung cancer	97	74.6
Breast cancer	24	18.5
Thyroid cancer	9	6.9
Previous therapy		
Previous surgery	102	78.5
Previous radiotherapy	58	44.6
Guidance mode		
Coplanar template guided	61	46.9
3D-PNCT guided	20	15.4
Simple CT guided	49	37.7
Tumor size		
≤4	55	42.3
>4	75	57.7

### Preoperative preparation and preplanning

All patients underwent blood routine, coagulation, and biochemical tests before SABT to rule out contraindications. The patient was secured on a CT scan bed and fixed in a custom-made vacuum bag in supine, prone, or lateral positions. Plain and contrast-enhanced CT scans were performed 1–2 days before treatment with a thickness of 5 mm. According to the requirements of the American Association of medical physicists (AAPM) , the image data is transmitted to BT-TPS (Beijing University of Aeronautics and Astronautics and Beijing Astronomical Technology Co. , Ltd.) for pre-planning (22, 23). The 90% gross tumor volume (GTV (GTV D90) dose should be as close as possible to the prescribed dose, while the organs at risk (OAR) dose should be as low as possible. The median prescription dose was 120 Gy (range, 100–160 Gy). The radionuclides used in the treatment were iodine 125 seeds (4.5 mm × 0.8 mm in size, with a half-life of 59.6 days; the activities of the seeds were 0.45–0.83 mCi (median 0.65 mCi).

### Stereotactic ablative brachytherapy protocol

The SABT protocol was as follows (24, 25): (1) the patient is placed on a CT simulator and fixed with a vacuum pad for a CT scan. (2) The body surface projection of the tumor target area was delineated, and local anesthesia and intercostal nerve block were performed. (3) Simple CT guided or use a coplanar or non-coplanar template to place the first pin on the body surface projection of the tumor as planned. The seed needles were all inserted into the target site; (4) A CT scan was performed to determine the exact position of the reference needle; (5) when the position of the reference needle did not match the predetermined position, the needle was adjusted in real time until the deviation was less than 2 mm; (6) the needle was inserted into the target area all at once; (7) the CT scan was repeated to confirm the position of all the tips and, in the same way, adjust for deviations of more than 2 mm; (8) the ^125^I seeds were delivered in a backward fashion with the Mick 200-TPV Applicator: TP transperineal (Mick Radio-Nuclear Inc., USA: Mount Vernon, NY); (9) The CT scan was performed again to confirm the distribution of ^125^I seeds in the targets. CT images were transmitted to BT-TPS for postplanning dose assessment. The patient will be discharged 1–2 days after SABT. All procedures follow the International Commission on Radiological Protection Recommendation (26). The dosimetric parameters, e.g., D90, were identified.

### Follow-up

The subjects were followed up by CT at the first month after SABT, every 3 months for 2 years, every 6 months for 3–5 years, and every year thereafter. The evaluation of tumor response was based on CT images after SABT.

### Statistical analysis

Statistical analyses were performed using SPSS 26.0 (IBM Corp., Armonk, NY, USA). The survival rate was estimated by the Kaplan–Meier method, univariate analysis by the logarithmic rank test, and multivariate analysis by Cox regression. P ≤ 0.05 was set as statistically significant.

## Results

### Patients

**Figure 1:**
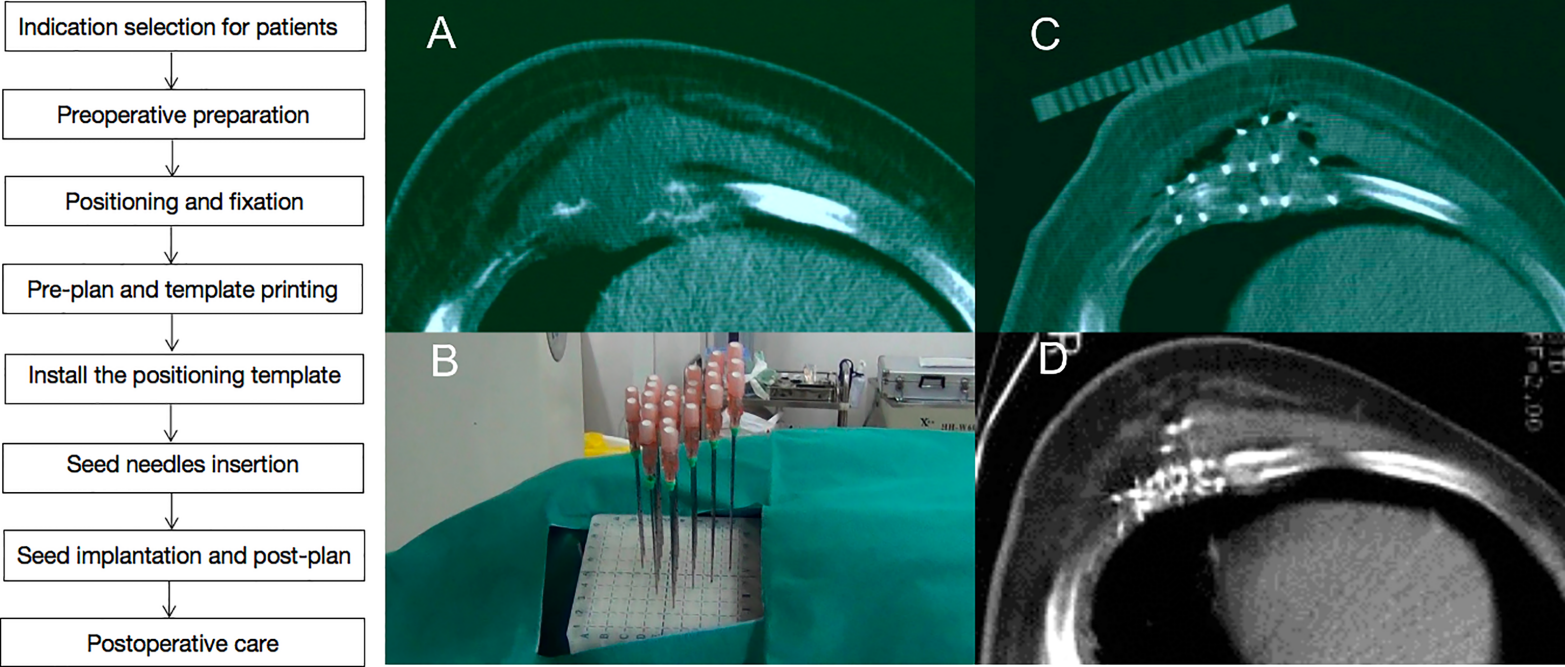
A case of recurrent chest wall cancer (rCWC) following surgery and adjuvant radiotherapy. **(A)** CT images of rCWC; **(B)** template-assisted CT-guided puncture the tumor target area; **(C)** ^125^I seeds were implanted as a salvage treatment; and **(D)** 3 months after the seed implantation, partial response was observed.

The 130 patients enrolled had a median age of 63 ± 11.7 years. A total of 59 patients died during a median follow-up of 22 (4–70) months; 71 patients survived (12 patients lost to follow-up since October 2020; **Figure 1**).

### Seed implantation

The median lesion diameter was 4.65 ± 1.61 cm (range, 1.5–8.9 cm). The median number of implanted seeds was 57 ± 20.65 (range, 23–128). The median seed radioactivity was 0.65 ± 0.07 mCi (range, 0.6–0.8 mCi). The median GTV D90 was 126 ± 15.27 Gy (range, 95–180 Gy).

### Treatment response

**Figure 2:**
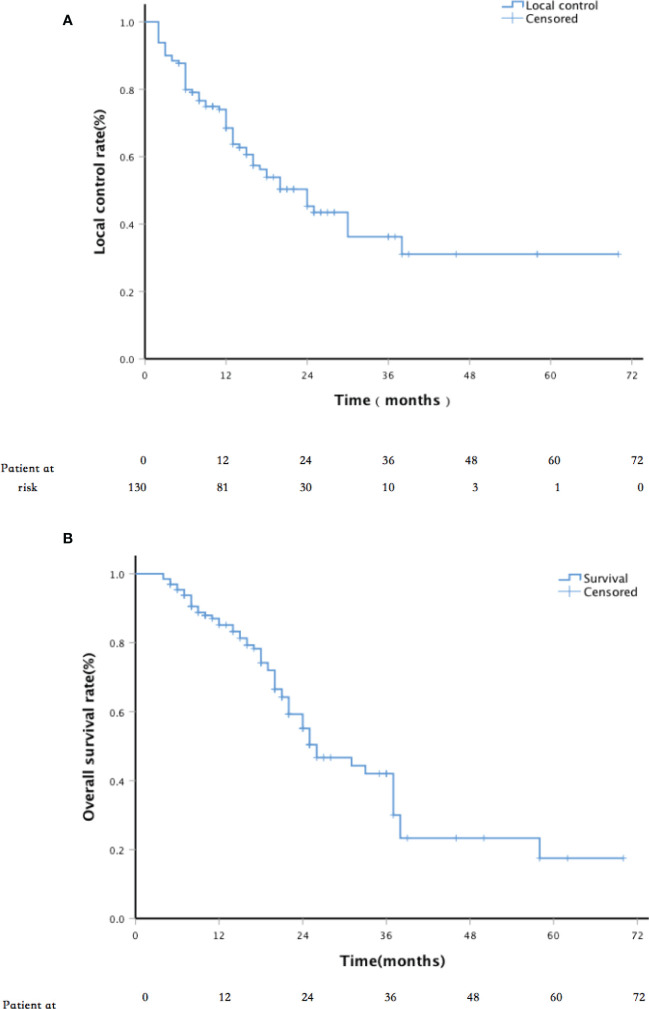
**(A)** Kaplan–Meier plots of local control (LC) and **(B)** Kaplan–Meier plots of survival.

**Figure 3:**
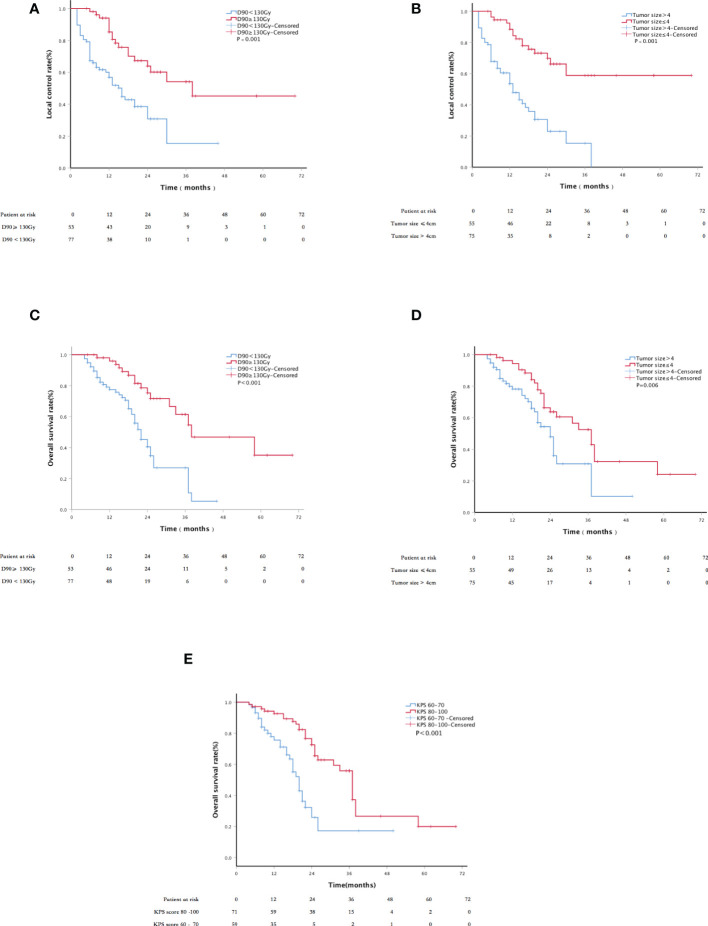
Kaplan–Meier curve about LC and survival: **(A)** the LC of patients with D90 ≥ 130 Gy and D90 < 130 Gy; **(B)** the LC of tumor size ≤4 cm and tumor size >4 cm; **(C)** the overall survival (OS) of patients with D90≥130 Gy and D90 < 130 Gy; **(D)** the OS of patients with tumor size ≤4 cm and tumor size >4 cm; and **(E)** the OS of patients with KPS scores 60–70 and 80–100.

The median follow-up period was 22 months (range, 4–70 months). The LC rates at 6, 12, 24, and 36 months were 88.3%, 74.3%, 50.4%, and 36.7%, respectively (**Figure 2**). Univariate analysis showed that sex, age, past surgical history, past radiotherapy history, implantation mode, pathological type, and physical status score were independent of LC (p = 0.119, 0.270, 0.993, 0.068, 0.550, 0.083, and 0.522, respectively). LC in patients with D90 ≥ 130 Gy was significantly better than that in patients with D90 < 130 Gy (p < 0.001). LC in patients with tumor size ≤4 cm was significantly better than that in patients with tumor size >4 cm (p < 0.001). In addition, multivariate analysis showed that the tumor size and postoperative D90 were independent prognostic factors for LC (**Figure 3**). The pain relief rate was 81%, and the median to remission time was 10 days.

### Survival

The 1-,2-, and 3-year survival rates were 85%, 56%, and 42%, respectively, with a median OS of 26 months (**Figure 2** **).** The 6-, 12-, 24-, and 36-month survival rates were 81.3%, 60.3%, 29.1%, and 20.3%, respectively. The median PFS was 13 months; 52.3% of patients had metastasis, 20.8% had local progression, and 26.9% had long-term LC. Univariate analysis showed that sex, age, past surgical history, past radiotherapy history, and implantation mode were not related to OS (P = 0.520, 0.111, 0.941, 0.178, and 0.099, respectively). Patients with D90 ≥ 130 Gy showed significantly better OS than patients with D90 < 130 Gy (p < 0.001). Patients with tumor size ≤4 cm had significantly better OS compared to patients with tumor size >4 cm (p = 0.006). Patients with good performance status (KPS ≥ 80) showed better OS than those with poor performance status (KPS < 80) (p < 0.001). Patients with the chest wall recurrence of breast cancer had longer OS than lung cancer (p = 0.005). Multivariate analysis showed that factors significantly associated with OS included the KPS score, tumor size, and postoperative D90 (all p < 0.001; **Figure 3**).

### Complications

There were 19 cases (14.6%) with grade I/II skin reaction and local skin pigmentation. No influencing factors were found to be associated with skin toxicity. No rib fracture, burst, pneumothorax, radiation pneumonia, and other adverse events occurred. There were no treatment-related adverse events of grade 3 or above.

## Discussion

This multicenter retrospective study presents the long-term follow-up results of SABT as salvage therapy. After a median follow-up of 22 months, patients showed relatively high rates of LC, OS, and improvement in the quality of life, particularly pain. The KPS score, tumor size, and postoperative D90 were significantly correlated with OS. The main complication was a mild skin reaction.

The management of rCWC after surgery and/or radiotherapy has been a challenging issue. EBRT as a salvage treatment option is promising for rCWC in previously exposed areas. However, challenges remain because it is difficult to deliver adequate doses to the target without affecting normal tissue, especially for tumors that had previously received full-dose therapy (27, 28). Hyperthermia combined with reirradiation or a high dose rate after loading is an effective treatment option in recent years (3, 29, 30). However, it is also limited by the tolerance of normal tissues, which often makes it difficult to increase the target dose, the overall efficacy is not satisfactory, and there are serious or even fatal treatment-related adverse reactions, such as ulceration, necrosis, chest fibrosis, and pneumonia (31, 32). SABT appears to be a viable alternative to adjuvant therapy. SABT may have the following advantages (9, 10): (1) compared with EBRT, the implanted radioactive seeds can irradiate the tumor continuously and without interval; (2) the radiation dose of the target can be increased high enough to achieve ablation effect; at the same time, the dose of correctly implanted seeds will rapidly decrease, thus not affecting normal tissue; (3) because of the minimally invasive nature of SABT, patients will soon recover and resume their daily lives after treatment.

For patients who develop recurrence in a previously radiated chest wall, the treatment options are more difficult. The overall clinical response rate was 38%–42.3% for radiation alone. There have been several small trials exploring reirradiation with the addition of local hyperthermia therapy. The 3-year LC rate was 25%. The overall clinical response rate was 60%–71% (33). Previous SABT data for rCWC patients were all obtained from single-center retrospective studies. Jiang et al. (16) reported 20 patients with refractory rCWC. The median follow-up time was 11.5 months. The 1-, 2-, 3- and 4-year tumor control rates were all 88.7%, respectively. The 1- and 2-, 3-, and 4-year OS rates were 53.3% and 35.6%, 35.6%, and 35.6% respectively. Shi et al. (17)reported 31 patients with a recurrent chest wall malignant tumor. The 6-month effective rate was 77.4%, and the LC rate was 83.9%. Jiang et al. (18) reported 19 cases of SABT guided by a three-dimensional (3D) non-coplanar template. The median follow-up time was 8 months. Complete response was observed in 18.1%, and partial response was observed in 59.1%; stable disease was observed in 8.1%, and the pain relief rate was 87.5%. Our study is consistent with those of previous studies, and higher LC rates were obtained. The 6- and 12-month LC rates were 88.3% and 74.3%, respectively. The chest wall recurrence tumor focus shallow, fixed, rich bone structure is conducive to 3D- printing non co-planar template (PNCT) fixation; the template is easier to meet the preoperative dosimetry requirements and can simplify the procedure; and the operator in a relatively short learning time can be skilled. The high rate of LC and the major causes of progression or death are metastasis to other sites suggest the need to explore and combine better systemic therapy to improve OS.

In terms of safety, 24%–33% of patients ≥grade 3 acute toxicity occurred using hyperthermia and radiation. The main adverse reactions were skin edema, ulceration and fibrosis (34), chest wall pain (neurogenic) (35), and rib fracture (36, 37); the incidence of moderate and severe skin ulcers was as high as 14% (38). The average duration of chest wall pain was 25 months (2–63 months), and 36% of patients never had relief from chest wall pain. A total of 34 (29%) of the 118 cases resulted in rib fractures with an average time of 22 months (3–46 months). The results of this study showed that the complications of SABT were mild and acceptable, mainly manifested as local skin pigmentation, without rib fracture, rupture, pneumothorax, radiation pneumonia, and other adverse events. No treatment-related adverse events of grade 3 or above occurred. Retreatment after radiotherapy remains a therapeutic challenge, but both the efficacy and toxicity of SABT are acceptable. There is even a history of radiation therapy similar to that of patients without radiation therapy. As a result, SABT has significant security advantages. The improvement of pain is good, the median onset time is short, and the quality of life of patients could be significantly improved.

This study has several limitations. First, as a multicenter study, the proportion of patients in different center groups is different, which may lead to potential bias. Secondly, the study was a one-arm retrospective study with 12 patients interviewed since October 2020, which may also lead to some bias. Thirdly, SABT serves as salvage therapy for the patients, but there is currently a lack of control group receiving standard salvage surgery or repeat EBRT. In addition, this group of patients is heterogeneous in the pathologic type, involving many different tumor species. There were no significant differences between lung cancer and breast cancer in the LC from SABT. Patients with the chest wall recurrence of breast cancer had better OS compared to patients with lung cancer. The sample size of different pathological types in the study group was quite different, and there might be some bias. However, this study was the first multicenter study including more than 100 patients to investigate the long-term safety and efficacy of SABT as salvage therapy in patients with rCWC. Therefore, randomized controlled prospective studies are warranted in the future.

## Conclusion

As salvage therapy following EBRT/surgery in patients with rCWC, SABT is safe and effective and has promising efficacy compared to historical data. Patients with a KPS score greater than 80, prescribed dose greater than 130 Gy, and tumor size less than 4 cm may bring better results.

## Data availability statement

The original contributions presented in the study are included in the article/**Supplementary Material**. Further inquiries can be directed to the corresponding authors.

## Ethics statement

The studies involving human participants were reviewed and approved by The Second Hospital of Tianjin Medical University. The patients/participants provided their written informed consent to participate in this study.

## Author contributions

Study concepts and design: SC, XuH, and JW. Literature research: BH and ZJ. Clinical studies: WY, YM, XiH, ZW, XZ, JD, HW, GC, RW,YS, and KZ. Experimental studies/data analysis: XiH, ZW, XZ, JD, and HW. Statistical analysis: ZJ and CH. Manuscript preparation: BH and CH. Manuscript editing: BH and ZJ. SC, XuH, and JW reviewed and edited the manuscript. All authors contributed to the article and approved the submitted version.
